# Unravelling the effect of droplet size on lipid oxidation in O/W emulsions by using microfluidics

**DOI:** 10.1038/s41598-024-59170-9

**Published:** 2024-04-17

**Authors:** Sten ten Klooster, Vincent J. P. Boerkamp, Marie Hennebelle, John P. M. van Duynhoven, Karin Schroën, Claire C. Berton-Carabin

**Affiliations:** 1 Laboratory of Food Process Engineering, Wageningen University and Research, Wageningen, the Netherlands; 2 Laboratory of Food Chemistry, Wageningen University and Research, Wageningen, the Netherlands; 3 Unilever Food Innovation Centre, Wageningen, the Netherlands; 4 Laboratory of Biophysics, Wageningen University and Research, Wageningen, the Netherlands; 5 INRAE, UR BIA, 44000 Nantes, France

**Keywords:** Monodisperse emulsions, Lipid oxidation, Microfluidics, Droplet size, Surfactants, Analytical chemistry, Imaging studies, Lab-on-a-chip, Microfluidics, NMR spectroscopy, Catalysis, Lipids, Fatty acids, Lipid peroxides, Oils

## Abstract

Lipid oxidation in emulsions is hypothesised to increase with decreasing droplet size, as this increases the specific oil–water interfacial area, where lipid oxidation is expected to be initiated. In literature, however, contradictory results have been reported, which can be caused by confounding factors such as the oil droplet polydispersity and the distribution of components between the available phases. In this work, monodisperse surfactant-stabilised emulsions with highly controlled droplet sizes of 4.7, 9.1, and 26 µm were produced by microfluidic emulsification. We show that lipid oxidation increases with decreasing droplet size, which we ascribe to the increased contact area between lipids and continuous phase prooxidants. Besides, a significant amount of oxygen was consumed by oxidation of the surfactant itself (Tween 20), an effect that also increased with decreasing droplet size. These insights substantiate the importance of controlling droplet size for improving the oxidative stability of emulsions.

## Introduction

Products containing polyunsaturated fatty acids (PUFAs) have a limited chemical stability due to lipid oxidation, which reduces the shelf-life by generating off-flavours and degrading the nutritional quality^1,2^. Lipid oxidation has become an increasing issue for the food industry given the current trend to promote omega-3 PUFAs in the diets, due to their health benefits. Concurrently, the use of additives, including synthetic antioxidants, tends to be reduced due to consumer preferences and potential health concerns^3^. Lipid oxidation issues are particularly marked in oil-in-water (O/W) emulsions, where lipid oxidation can be initiated at the oil–water interface by contact between the lipid substrate and water-soluble prooxidants, such as metal ions^4,5^.

Recent publications have shed light on the spatiotemporal aspects of lipid oxidation in O/W emulsions, which has led to hypotheses that need to be experimentally substantiated^6–9^. One spatiotemporal aspect that is poorly understood is how the droplet size affects lipid oxidation^4^. A logical hypothesis would be that smaller droplets would oxidise faster because they have a larger specific interfacial area (m^2^ interface/m^3^ oil), allowing for more initiation reactions. Some results in literature accordingly confirmed that small droplets oxidise faster than large ones^6,10–19^, whereas others indicated that larger droplets oxidise faster^17–25^, or that the droplet size does not influence lipid oxidation^6,15,16,18,24,26–29^. Whether small droplets or large droplets oxidise faster has been argued to be dependent on the components present, such as antioxidants, emulsifiers, and oil^6,17,19^. Additionally, the applied incubation conditions, such as the storage temperature^18^, have been argued to influence whether small or large droplet oxidise faster.

Part of the confusion in the literature about how the droplet size influences the lipid oxidation rate is expected to be related to the difficulty to vary the droplet size univocally. Often, other parameters are concurrently changed, which may affect lipid oxidation as well. For example, the emulsification procedure may influence lipid oxidation in emulsions^15,18,30^. Possible explanations include the production of free radicals by certain processes that induce cavitation^31^, or the modification of the oil–water interfacial composition^19,32^. The emulsification procedure and conditions modulate the amount of unadsorbed emulsifiers and the partitioning of other surface-active molecules, such as certain antioxidants^4^. Furthermore, this leads to different droplet size distributions, and since we hypothesise faster oxidation of smaller lipid droplets, it can also be expected that the droplet size polydispersity greatly influences the lipid oxidation rate^33–35^. Moreover, in polydisperse emulsions the smallest droplets oxidise fastest^34^. Thus, when using different emulsification procedures, the inherent variation of multiple parameters obscures the actual effect of the droplet size on lipid oxidation.

Only a limited number of studies have attempted to address the effect of the specific surface area on lipid oxidation by deploying approaches involving a fine control over the droplet size and size distribution. One notable example is the work of Azuma et al.^17^, who employed microfluidic emulsification to generate monodisperse emulsions with varying droplet sizes that served to distinguish between two types of oils while using three different emulsifiers. Their findings revealed an accelerated oxidation of smaller droplets when soybean oil was used as the dispersed phase, whereas the opposite trend was observed when fish oil was used as the dispersed phase. The oil concentration in these emulsions was low (0.5%), leading to a high oxygen-to-oxidisable substrate ratio, and thus also to a very high conversion of the oxidizable substrate. Another interesting paper was recently published by Li et al.^10^, who utilized flow cytometry to investigate the impact of droplet size on lipid oxidation within a single emulsion that contained a wide range of droplet sizes. The authors also concluded that smaller droplets oxidise faster.

To advance the understanding of the effect of droplet size on lipid oxidation in emulsions, an essential first step is to very precisely control the droplet size by using microfluidic devices, as was the case in the work of Azuma^17^. Here, we used upscaled microfluidic emulsification devices to prepare highly monodisperse emulsions with three distinct droplet sizes, all of them with the same oil volume fraction and emulsifier^36^. We thereby systematically varied the droplet size and, accordingly, the total oil–water interfacial area. This work can thus contribute to pinpointing specifically how and to which extent the droplet size and interfacial area can influence lipid oxidation in emulsions, and thus lead to a better understanding of the overall course of this reaction in such matrices.

## Results and discussion

### Emulsion production

**Figure 1:**
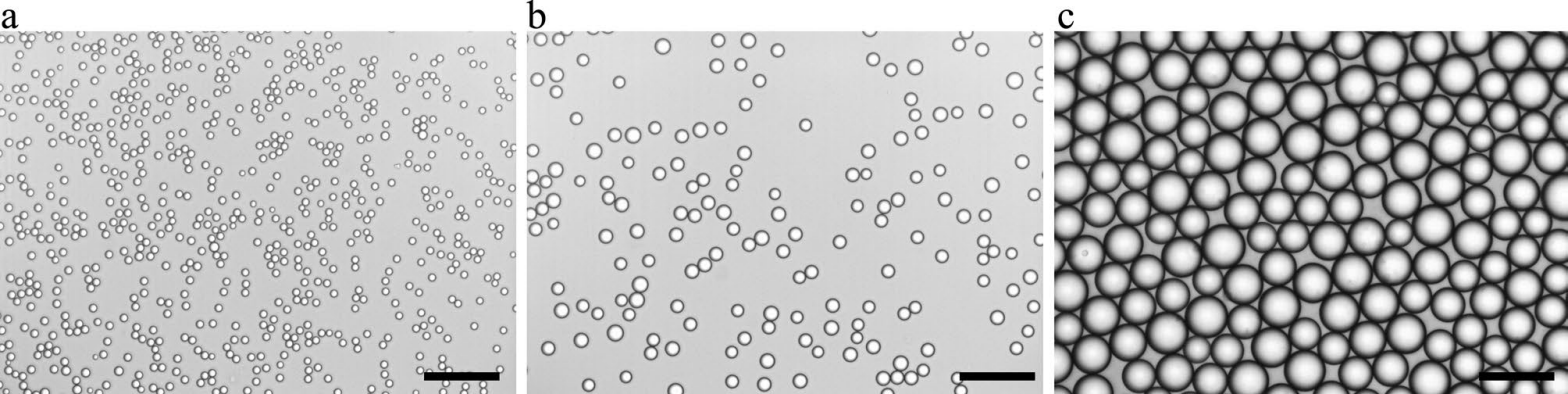
Light microscopy images of rapeseed oil droplets in a continuous phase containing 2 wt.% Tween 20, which were prepared with microfluidic emulsification. Three different droplet sizes were prepared: small (D_1,0_ = 4.7 µm, *A* = 1.37 m^2^/g oil) (**a**), intermediate (D_1,0_ = 9.1 µm* A* = 0.70 m^2^/g oil) (**b**), and large droplets (D_1,0_ = 26.0 µm, *A* = 0.24 m^2^/g oil) (**c**). Droplet size distributions are shown in Fig. S2. Light microscopy images of replicate emulsions are shown in Fig. S3. Scale bar represents 50 µm.

The present study investigated three distinct emulsions with contrasted droplet sizes, and each emulsion was produced in independent duplicates. The emulsions with small droplets had an average droplet size of 4.5 and 4.8 µm (coefficient of variation (CV): 7.3 and 6.6%); the emulsions with intermediate droplets had an average droplet size of 8.9 and 9.3 µm (CV: 10.8 and 7.8%); and the emulsions with large droplets had an average droplet size of 25 and 27 µm (CV: 5.8 and 12%) (Fig. 1, S2 and S3). The difference in diameter between the smallest and largest droplets was thus a factor six. The fraction of interfacial Tween 20 was estimated to be minor (< 2% of the total Tween 20 used), implying that ~ 98% of the used surfactant remained in excess in the continuous phase (Table S1), for all emulsions^4,37^. Just after emulsification, the hydroperoxide concentration was 1.0 ± 0.4 mmol/kg oil, with no significant difference (*p* > 0.05, one-way ANOVA) between the different droplet sizes.

### Lipid oxidation in droplets of different sizes

**Figure 2:**
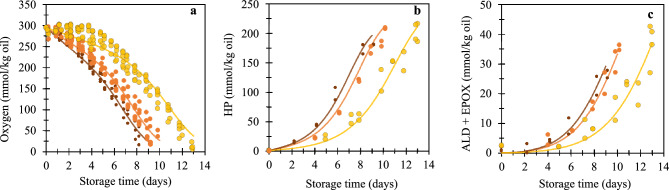
Headspace oxygen consumption (**a**), lipid hydroperoxide formation (**b**), and secondary oxidation product formation (**c**) upon incubation of the emulsions at 25 °C. Symbols correspond to emulsions with different droplet sizes: small droplets (D_1,0_ = 4.7 µm) (small brown circles), intermediate droplets (D_1,0_ = 9.1 µm) (intermediate orange circles), and large droplets (D_1,0_ = 26.0 µm) (large yellow circles). The lines are drawn to guide the eye based on the model by Schroën et al.^38^ (Section 2.5 and 5.1). Both dependent and independent replicates are shown as individual data points.

The monodisperse emulsions were incubated at 25 °C in the presence of an iron-EDTA initiator. Under these incubation conditions, samples were physically stable over two weeks (Fig. S4). Over incubation, all emulsion samples consumed almost all of the available oxygen in the headspace, and reached high levels of lipid oxidation products (Fig. 2). Oxygen consumption, and the formation rate of hydroperoxides, aldehydes, and epoxides all increased with decreasing droplet size (Fig. 2). When looking in further detail, NMR spectroscopy enabled us to classify oxidation products based on their fatty acid precursor (i.e., oleic, linoleic, and linolenic acids). The contribution of each fatty acid to the formation of oxidation products was found to be independent of the droplet size (Fig. S5).

1 $${\text{LOOH}} + {\text{Fe}}^{3 + } \to {\text{LOO}}^{ \cdot } + {\text{H}}^{ + } + {\text{Fe}}^{2 + }$$

2 $${\text{LOOH}} + {\text{Fe}}^{2 + } \to {\text{LO}}^{ \cdot } + {\text{OH}}^{ - } + {\text{Fe}}^{3 + }$$

3 $${\text{LOOH}}\left( {{\text{heat}}/{\text{light}}} \right) \to {\text{LO}}^{ \cdot } + {\text{OH}}^{ \cdot }$$

4 $${\text{LO}}^{ \cdot } /{\text{LOO}}^{ \cdot } + {\text{LH}} \to {\text{LOH}}/{\text{LOOH}} + {\text{L}}^{ \cdot }$$

5 $${\text{L}}^{ \cdot } + {\text{O}}_{2} \to {\text{LOO}}^{ \cdot }$$

6 $${\text{LOO}}^{ \cdot } + {\text{LOO}}^{ \cdot } \to {\text{nonradical products}}$$

Especially at the beginning of incubation, hydroperoxide formation was more rapid for the small droplets compared to the intermediate and large droplets (Fig. 2b). This result may be ascribed to the increased specific interfacial area that favours contact between the prooxidants in the aqueous phase and the lipid substrate. Hydroperoxide decomposition results in peroxyl and alkoxyl radicals (Reactions 1 and 2) that propagate the lipid oxidation reaction (Reactions 4 and 5), whereas at low residual oxygen the hydroperoxide formation levelled off (Reaction 4 and 5). A faster onset of lipid oxidation for small droplets was previously reported for fish oil-enriched mayonnaise^11^.

The formation of secondary oxidation products also increased with a decreasing droplet size (Fig. 2c), although this was, in absolute values, less pronounced compared to the increase in hydroperoxides and decrease in oxygen concentration. Therefore, the specific surface area does not seem to affect the formation of secondary oxidation products directly. Indirectly, the higher concentration of primary oxidation products causes a faster formation of secondary oxidation products for smaller droplets (see modelling section in the supporting information). This is in line with a previous study, where it was found that a similar reaction rate constant for aldehyde formation could fit well the experimental results obtained in emulsions stabilised by different emulsifiers^38^.

### Additional effects

**Figure 3:**
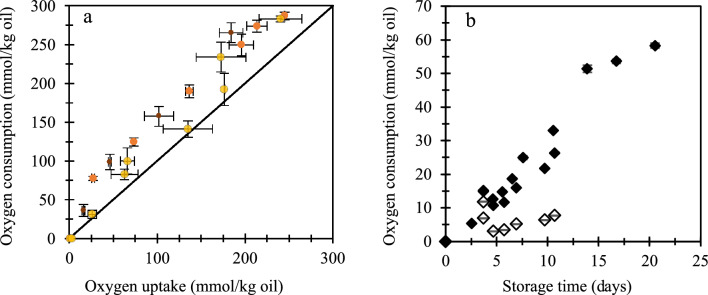
(**a**) Measured oxygen consumption against calculated oxygen uptake in emulsions with small droplets (D_1,0_ = 4.7 µm) (small, brown circles), intermediate droplets (D_1,0_ = 9.1 µm) (intermediate, orange, circles), and large droplets (D_1,0_ = 26.0 µm) (yellow, large circles). Oxygen consumption was measured from the oxygen content in the tubes’ headspace, whereas oxygen uptake was calculated from the detected oxygen-bearing lipid oxidation products (hydroperoxides, aldehydes, and epoxides). The solid line is the parity line: y = x, and it represents oxygen consumption equals oxygen uptake. (**b**) Oxygen consumption by a 2-wt.% Tween 20 solution (open diamonds) and by a 2-wt.% Tween 20 solution with 0.19 mM iron-EDTA (filled diamonds). Despite the absence of oil, in panel (**b**) oxygen consumption by Tween 20 is expressed by assuming an oil content of 10 wt.% to allow for direct comparison of the obtained values with those obtained in the emulsion systems. Error bars (sometimes within markers) denote standard deviations of three measurements on three individually incubated samples.

We found that the molar amount of oxygen consumed from the headspace exceeded the molar amount of oxygen incorporated as lipid oxidation products (Fig. 3a), which indicated that additional oxygen consumption had taken place. This is in contrast to findings in previous work, where a closed oxygen balance for the oxidation of mayonnaise and bulk oil was found when applying the same methods to quantify oxygen consumption and lipid oxidation products as used in the present work^39^. In the present case, we used a substantial amount of Tween 20 to stabilise the emulsion, which is known to oxidise as well^40–42^. Thus, a possible explanation for the observed discrepancy could be the contribution of Tween 20 oxidation. It is difficult to find evidence in the literature for the stoichiometry of such a reaction, and thus to evaluate the potential contribution to the overall oxygen consumption. One previous study examined lipid oxidation, including oxygen consumption in Tween 20-based emulsions; however, in this example^37,39^, a much lower Tween 20-to-oil ratio was used compared the present work (12-fold lower ratio), which implies that the relative effect of Tween 20 oxidation compared to lipid oxidation was probably much smaller, and thus not pinpointed.

To test our hypothesis, we incubated the continuous phase, without oil, in the same conditions as used for the emulsions (i.e., 2 wt.% Tween 20 with or without 190 µM iron-EDTA). In the presence of iron-EDTA, we observed an oxygen consumption of ~ 30 mmol (per kg equivalent oil) over 11 days, and of ~ 60 mmol/kg equivalent oil over 20 days (Fig. 3b). This amount is somewhat lower than the differences shown in Fig. 3a (maximum difference was ~ 75 mmol O_2_/kg oil), which could suggest that Tween 20 oxidised faster—and to a higher extent—in an oxidising emulsion than when incubated without oil. This could be explained by the presence of radical species in the oxidising emulsions, as reactive free radicals have been shown to be able to abstract hydrogen atoms from polysorbates.^40–42^ Once initiated, Tween 20 oxidation might propagate oxidative reactions to both Tween 20 and TAGs (Reactions 4 and 5).^43^ Although oxidation of Tween 20 reduces the amount of oxygen available to oxidise the lipid substrate, small droplets still oxidise faster (Fig. 2b).

Finally, another side effect that is worth mentioning when making such molar balances is the fact that some oxygen diffused into the tubes upon incubation (Fig. S6). The oxygen ingress rate depends on the oxygen concentration gradient present (classically described by Fick’s law). We have incorporated the oxygen ingress into the kinetic model based on diffusion rates measured under maximum oxygen gradient (i.e., full oxygen depletion inside container) (Supporting information section Introduction, Fig. S6). This side-effect did not affect our conclusion that small droplets oxidise faster than large ones because the contribution from oxygen diffusion into the tubes is relatively small, especially at the beginning of incubation, when the effect of droplet size on the lipid oxidation rate was largely predominant (Fig. 2).

### Contradicting views from literature put into perspective

In line with our results, a number of other studies have also reported that small droplets oxidise faster than larger ones^6,10–19^. Multiple hypotheses were proposed to explain this. In some hypotheses, the droplet size is used as a proxy for the interfacial area that determines the area for (a) influx of oxygen^16^, (b) partitioning of emulsifiers and other surface-active molecules^4^, (c) and reactivity (this work)^11^. Hypothesis (a) of a stimulated oxygen diffusion is unlikely in a continuously mixed system as used in our work because the diffusion of reactants is much faster in these emulsions than the reaction rate. As shown by Schroën et al., mass transfer greatly exceeds reaction effects when using droplets of up to 100 µm (Damköhler II number 10^−7^–10^−5^ for droplets of 1 µm)^38^. Hypothesis (b) can affect lipid oxidation but only when the variation in the amount of interfacial area substantially modifies the concentration of emulsifier present in the continuous phase. An increased amount of such emulsifiers (proteins or surfactants, including Tween 20) in the continuous phase usually promotes their antioxidant effect^37^. In our study, the fraction and concentration of unadsorbed Tween 20 was similar for all three emulsions (at least 98% of the total Tween 20 amount) (Table S1), and is therefore not expected to have played a differentiating role. In a study by Horn and co-workers, a difference in the amount of unadsorbed emulsifiers could have played a role (Table 1) in promoting lipid oxidation for small droplets, compared to large droplets^19^. In the current work, we hypothesise (c) that the faster oxidation of small droplets is caused by their larger specific interfacial area, which promotes lipid oxidation initiation reactions at the interface by the water-soluble iron-based catalyst.

**Table 1:** Summary of literature that describes the influence of the lipid droplet size on lipid oxidation.

Article	Oxidation rate	Oil (% of emulsion)	Emulsifier (wt.% of continuous phase)	Other components (wt.% of emulsion)	T (°C)	Method(s)	Droplet sizes (µm)
^10^	S > L	0.15% Sp. Soybean, 0.05% BN oil	0.14% SDS	0.1% Xanthan	37	RS	2–8, > 20
^12^	S > L	?% DHA	1% DG MS	0.5% Xanthan	25	ME	D? 3.4, 6.4
^13^	S > M > L	28% Sp. sunflower oil	2% BSA	0.04% SA	47	RS (L), and HP (M,S)	D[2,3] 0.4, 1.2, 8.3
^11^	S > M > L	16% fish oil, 64% rapeseed oil	20% Egg yolk	Mayo system	37	Combinator (and viscorotor)	D[2,3] 2.5, 3.2, 3.3
^14^	S > L	28% Sp. sunflower oil	2% BSA	0.04% SA	37/47	RS (L) and HP (S)	D[2,3] 0.36, 1.2
^15^	S > M1 = M2 > L	27% flaxseed oil	5% WPI	0.02% SA	25	RS and HP	D[3,4] Between 0.46–2.2
^16^	S > M > L	1% Methyl α-linolenate	0.15% ML750	0.01% SA	55	RS and ME	D[2,3] 1.4, 2.8, 7.4, 31
^17^	S > M > L	0.5% Sp. Soybean oil	0.2% PG / SG FA ester	3 mM AAPH + 20 µM Fe^2+^	37	MF	D? 6.4, 11, 38
^18^	S > L	9.2% Fish oil	1% ML750	0.02% SA	5	ME (S)/MF (L)	D[2,3] 26.8, 1.2
^6^	S > M > L	78% Sp. soybean oil	23% Egg yolk	Mayo system	30	RS	1–4
^19^	S > L	10% Fish oil	1% WPI	0.02% SA	20	RS and HP	D[2,3] 0.26, 0.71
^19^	L > S	10% Fish oil	1% NaCas + β-lac	0.05% SA	20	RS and HP	D[2,3] 0.36, 0.55
^23^	L > M > S	0.5% Fish oil	Cas + WP	–	–	RS and HP	D[2,3] 0.70, 0.89, 1.3
^17^	L > M > S	0.5% Sp. Fish oil	0.2% PG FA ester / SG FA ester	3 mM AAPH + 20 µM Fe^2+^	37	MF	D? 7, 11, 38
^24^	L > S	18% Sp. sunflower oil	1.5% WPI	–	50	RS (L), and HP (S)	D[2,3] 1.1, 51
^20^	L > S	20% Camelina oil	3% NaCas	0.02% SA	60	HP	D? 0.19, 0.29
^21^	L > S	10.6% Linoleic acid	0.5, 1, 2, 3, 4, 7, 9.5% WPI / NaCas	0.02% SA	50	RS (L), and HP (S)	D[2,3] 0.31, 0.65
^22^	L > M > S	10% Sp. (silica) Soybean / fish oil	0.9% SG lauryl ester / ML750	100 µM EDTA	40	ME	D? ~ 0.8, ~ 3.3, ~ 10
^18^	L > M = S	9.2% Fish oil	1% ML750	0.02% SA	30	HP	D[2,3] 0.09, 0.28, 0.59
^18^	L > S	9.2% Fish oil	1% ML750	0.02% SA	30	ME (S)/MF (L)	D[2,3] 27, 1.2
^18^	L = M = S	9.2% Fish oil	1% ML750	0.02% SA	5	HP	[D3,2] 0.09, 0.28, 0.59
^6^	S = M = L	78% (Sp.) soybean oil	23% Egg yolk	Mayo system (+ ascorbic acid)	30	RS	1–4
^24^	S = L	18% (Sp.) sunflower oil	1.5% WPI	(1 mM Rutin)	50	RS (L), and HP (S)	D[2,3] ~ 1, ~ 50
^27^	S = M = L	30% Sunflower oil	1% NaCas / Tween 20 / NaCas + Tween 20	–	60	RS and HP	D[2,3] 0.67, 0.84, 1.4, 3.2
^26^	S = M = L	30% Sunflower oil	3% WPC	0.2% SA	35	RS and HP	D[2,3] 0.51, 0.63, 1.2, 1.5, 1.9
^16^	S = M = L	Methyl linoleate	ML750 (0.15% w/v)	0.01% SA	55	ME	D[2,3] 1.5, 2.6, 6.5, 20, 29
^28^	S = L	10/30% Canola oil / caprylic acid	0.5% WPI / SG ester	0.01% SA	50	HP	D[2,3] (0.26 and 1.1), (1.5 and 1.1)
^15^	S > M1 = M2 > L	27% flaxseed oil	5% WPI	0.02% SA	25	RS and HP	D[3,4] between 0.46–2.2
^29^	S = L	10% sp. Fish oil	0.5% Tween 80	(0.002% gallic acid)	25 / 35	RS (L), and HP (S)	D? ± 0.075, ± 0.3

S = small droplets, M = medium/intermediate droplets, L = large droplets, Sp. = stripped, DHA = Docosahexaenoic acid, BN = brominated, WP(I)(C) = whey protein (isolate) (concentrate), PG = polyglycerol, SG = sugar, ML-750 = decaglycerol monolaurate, (Na)Cas = (sodium) caseinate, B-LG = β-lactoglobulin, DG MS = decaglycerin monostearate, TP = tocopherol, SA = Sodium azide, ME = membrane emulsification, MF = microfluidic emulsification, RS = Rotor stator, HP = high pressure, ‘?’ means unknown how the weighting for the average droplet size was done. If a emulsion preparation method is stated in brackets, then this method was, or was not, used to make the large and small droplets. If a component is between parentheses, then this means that the effect of droplet size on lipid oxidation was performed studied with and without this component. Table S1. Calculation of the ratio of interfacial Tween 20 to total Tween 20 for the small droplets (D_1,0_ = 4.7 µm).

Other studies have found that larger droplets oxidised faster than smaller ones^17–24^, or that droplet size did not affect lipid oxidation in emulsions^6,16,24,26–28^. These results may have been caused by co-effects that could obscure the individual effect of droplet size on lipid oxidation. These effects include, but are probably not limited to: (i) the ratio between pro-oxidants and interfacial area, (ii) metal-chelation, (iii) elevated incubation temperatures, (iv) different emulsification method, (v) oil type, and (vi) too limited differences in droplet sizes. We will now shortly discuss all these factors.

(i) If the ratio of prooxidant to interfacial area is low and if the prooxidants are attracted to the interface (e.g., a negatively charged interface attracts metal cations), then the interfacial area is most likely non-limiting for the initiation of lipid oxidation, and thus creating more interface is not expected to lead to favoured initiation reactions.
(ii) When prooxidants are scavenged by a large excess of metal chelators in the continuous phase, the initiation of lipid oxidation is not expected to take place at the interface anymore, and non-interfacial-related initiation reactions may become dominant (e.g., the decomposition of LOOHs by heat (Reaction 3) or the formation of singlet oxygen by light). In that case, we expect that the lipid oxidation rate is not influenced by the droplet size.
(iii) If lipid oxidation is initiated at elevated temperature (above 40–60 °C), the heat-catalysed decomposition of hydroperoxides (Reaction 3) could start playing a role, which is unrelated to the specific interfacial area. This can explain why studies carried out at high temperatures often indicate that droplet size does not affect lipid oxidation (or even that larger droplets oxidised faster than smaller droplets) (Table 1).
(iv) The use of different emulsification methods can in itself influence lipid oxidation. For example, by cavitation, free radical formation will be promoted, which can in turn impact the formation of lipid oxidation products over time^4,44^. The emulsification method can also alter the interfacial composition^19,23,32^, which has been reported to lead to better oxidative protection for the smaller droplets in specific emulsions^23,32^. The authors of this work suggested that the interfacial composition is more important than the amount of specific interfacial area^23^. The emulsification procedure can also affect the amount of very small droplets (10–200 nm) that are formed^34^. These very small droplets are hardly detected by commonly used droplet size characterisation methods, but have been shown to be highly reactive. This highlights the importance of prevention of the formation of such droplets, e.g., by using microfluidics.
(v) Azuma and co-workers showed that with stripped soybean oil, an emulsion with relatively small droplets oxidised faster, whereas with stripped fish oil, the emulsion with relatively large droplets oxidised faster^17^. This was ascribed to the molecular conformation of fish oil lipids (i.e., long chain PUFAs such as docohexaenoic acid, DHA) at the oil–water interface. It was previously reported that when oxidizing PUFAs present as micelles dispersed in an aqueous phase, increasing the FA unsaturation degree, especially with omega-3 PUFAs, increased the oxidative stability^45^. This effect was repeatedly found, which led to proposing the so-called paradox of omega-3 PUFA oxidation, and it has been linked to a packed micellar conformation of the PUFA with the surfactant^46,47^. As can be seen from Table 1, in some fish oil-based emulsions, larger droplets indeed oxidised faster than smaller ones (or there was no effect); yet, in other studies, emulsions with smaller fish oil droplets oxidised faster (Table 1), making it difficult to draw generic conclusions. In the current work, when comparing the relative levels of oxidation of oleic, linoleic, and linolenic acid, it is clear that these levels remained constant over time and were independent of the droplet size (Fig. S5).
(vi) Our results indicate that in the conditions presently used, a large difference in oil droplet size is needed to observe an effect of this parameter on lipid oxidation. If the droplet sizes were only slightly different from one emulsion to another (Table 1), or if the distributions largely overlap, the effect that the specific interfacial area has on lipid oxidation might be easily obscured by other potentially interfering factors, as described above. This implies that it is essential to measure the droplet size distribution to determine the interfacial area accurately, which requires specific efforts and experimental approaches for emulsions with polydisperse and sometimes even multimodal droplet size distributions.

In order to truly differentiate these effects, preparation of defined reaction systems is needed. Thanks to the highly monodisperse emulsions prepared in this study, we were able to untaintedly assess the effect of droplet size on lipid oxidation. In future work, the data presented here will be of paramount importance for extensive modelling on how the droplet size influences lipid oxidation.

In summary, we systematically studied the effect of droplet size on lipid oxidation in Tween 20-stabilised monodisperse O/W emulsions, which were prepared with microfluidic emulsification devices. The increased oxidation of smaller droplets was ascribed to their larger amount of oil–water interface, where lipid oxidation was initiated by an iron-based oxidation catalyst. The fast lipid oxidation for small droplets also favoured Tween 20 oxidation. This could be a result of higher concentrations of radicals in small droplets, which may not just propagate lipid oxidation within the droplets, but also Tween 20 oxidation at the interface and possibly even in the continuous phase of the emulsion. Even though many other factors influence the oxidative stability of an emulsion, these insights show that it is important to also consider the droplet size for improving our insights in the oxidative stability of emulsion products and, ultimately, to prepare emulsion products with a high oxidative stability.

## Materials and methods

### Materials

Rapeseed oil was kindly provided by Unilever (Wageningen, the Netherlands) and stripped with alumina powder (MP EcoChromet ALUMINA N, Activity: Super I, Biomedicals) to remove surface-active impurities and endogenous antioxidants, in particular tocopherols^48^. The fatty acid composition of the rapeseed oil included: 68% oleic, 17% linoleic, 8% linolenic, and 7% saturated fatty acids. Tween 20 (also known as polysorbate 20), ethylenediaminetetraacetic acid calcium disodium salt (EDTA), iron(II) sulfate heptahydrate (FeSO_4_), sodium phosphate monobasic dihydrate, and sodium phosphate dibasic dihydrate were purchased from Sigma-Aldrich (Zwijndrecht, the Netherlands). For cleaning of the microfluidic chips, we used ethanol (purity 96% v/v, VWR International B.V., Amsterdam, the Netherlands) and piranha solution, which is a 3:1 v/v ratio of 98% sulphuric acid and 35% hydrogen peroxide (both obtained from Sigma-Aldrich, Zwijndrecht, the Netherlands). Ultrapure water (18.2 MΩ) was used for all experiments and prepared using a Milli-Q system (Millipore Corporation, Billerica, MA, USA). The assay reagent for measuring the triglyceride (TAG) content and a TAG standard were purchased from HUMAN Gesellschaft für Biochemica und Diagnostica mbH (Wiesbaden, Germany). The assay reagent content was: 50 mmol/L PIPES buffer (pH 7.5), 5 mmol/L 4-chlorophenol, 0.25 mmol/L 4-aminoantipyrine, 4.5 mmol/L magnesium ions, 2 mmol/L ATP, 1.3 U/mL lipases, 0.5 U/mL peroxidase, 0.4 U/mL glycerol kinase and 1.5 U/mL glycerol-3-phosphate oxidase. n-Hexane (97%) and 2-propanol (99.8%) were obtained from Actu-All Chemicals (Oss, the Netherlands). Deuterated chloroform (CDCl_3_) with 0.03% tetramethylsilane (TMS), deuterated dimethylsulfoxide (DMSO-*d*_6_), and deuterated 4 Å molsieves were purchased from Eurisotop (Saint-Aubin, France).

### Microfluidic emulsion preparation

#### Preparation of the continuous phase

The day before emulsion production, Tween 20 (2 wt.%) was dissolved in phosphate buffer (pH 7.0, 10 mM) and stirred for 30 min at room temperature. Both the dispersed phase (stripped rapeseed oil) and the continuous phase (2 wt.% Tween 20 in phosphate buffer) were filtered using a 0.22-µm regenerated cellulose filter (Minisart High-Flow, Sartorius Stedim Biotech GmbH, Goettingen, Germany) prior to use to prevent any dust from entering the microfluidic chip.

#### Microfluidic chip

To produce highly monodisperse emulsions, microfluidic devices called UPE (Upscaled Partitioned EDGE [Edge-based Droplet Generation]) were used (Fig. S1)^36^. These glass chips were designed in our lab and produced by deep reactive ion etching by Micronit Microfluidics (Enschede, The Netherlands). To make the smallest droplets (~ 4.5 µm), a chip with 11,088 droplet formation units (DFUs) of 5 × 1 µm (width × height) each was used. To produce the intermediate (~ 9 µm) and largest droplets (~ 26 µm), a chip with 8064 DFUs of 10 × 2 µm (width ⨯ height) each was used. In the latter case, a higher dispersed phase pressure was applied to produce 26-µm droplets. Information about the microfluidic chip characteristics, the principle, and regimes of oil droplet formation can be found in previous work^36,49,50^.

#### Microfluidic chip cleaning

The chips were cleaned and operated as described previously^36^. In brief, after finishing an experiment, the chip (including the plateaus) was flushed with ethanol, followed by sonication (Branson 1800, Brookfield, CT, USA) in ethanol for 90 min and then in water for 10 min. Next, the chip was placed in an oven at 500 °C for 2 h. The day before an experiment, the chip was sonicated in a glass beaker with piranha for 90 min, which was followed by sonication in ultrapure water for 90 min. It was stored overnight in ultrapure water.

#### Emulsion preparation by microfluidics

On the day of the experiment, the cleaned microfluidic chip was placed in a chip holder from Micronit (Fluidic Connect PRO Chip Holder with 4515 Inserts, Micronit Microfluidics, Enschede, The Netherlands). The channels were first rinsed with ultrapure water and then with the continuous phase. Next, the dispersed phase channel was rinsed with an excess amount of dispersed phase, after which the dispersed phase channel outlet was blocked. The pressure over the dispersed phase was increased to let the dispersed phase flow through the shallow connection between the dispersed and continuous phase channel, the so-called plateau. The plateau splits into many micro-plateaus (Fig. S1). At the location where the micro-plateaus meet the relatively deep continuous phase channel, oil droplets were generated spontaneously. Increasing the pressure over the dispersed phase increased the droplet formation frequency without changing the droplet sizes. When the pressure was increased beyond the transition pressure, larger monodisperse droplets were formed (see Section "Additional effects" for droplet sizes). Based on the production in previous work^36^, the continuous phase flow was set such that the resulting oil concentration of the emulsion was > 10 wt.%. The emulsion was collected in a beaker overnight for the small and intermediate droplets and over 3 h for the large droplets. To prevent lipid oxidation and evaporation of the continuous phase as much as possible during emulsion production, the headspace of the collection beaker was flushed with a continuous flow of water-saturated nitrogen gas. Finally, the oil content was adjusted to 10 wt.% by addition of continuous phase after the determination of the triglyceride content in the collected emulsion (Section "Additional effects"). The emulsions were incubated directly after production as described below.

#### Incubation and sample taking

An equimolar mixture of FeSO_4_ and EDTA was used as an oxidation initiator, which can fairly represent oxidation pathways in food products that would contain (traces of) metal ions^51^. The mixture was prepared by separately dissolving 12 mM FeSO_4_ and EDTA in a phosphate buffer (pH 7.0, 10 mM). Equivalent volumes of each solution were mixed, and the iron-EDTA complex was allowed to form under moderate stirring in the dark for exactly 1 h.

To 1.5-mL polypropylene tubes (total volume ~ 1.75 mL), 12.5 µL of the iron-EDTA complex solution and 387.5 µL of emulsion were added, so that the final concentration of FeSO_4_ and EDTA in the emulsion was 190 µM. The samples were incubated, in the dark, in heating blocks, at 25 °C, and shaken at 800 rpm to prevent creaming. Samples were taken at selected time points based on the remaining oxygen content in the headspace (Section "Contradicting views from literature put into perspective"). The samples were covered with a nitrogen blanket and stored at − 80 °C for 48 h to two weeks, before further extraction and lipid oxidation analyses were performed.

The oxidation of Tween 20 was assessed by incubating aliquots of the surfactant solution (aqueous phase), without oil. Hereto, tubes (1.5 mL) were filled with 387.5 µL of Tween 20 solution (2 wt.%) in either the presence or absence of 12.5 µL of the iron-EDTA complex solution. Both samples were incubated as described above and oxidation was quantified over time by the decrease in headspace oxygen content.

### Emulsion characterisation

#### Triglyceride content

The triglyceride (TAG) content in the obtained emulsions was measured using a colorimetric method^52,53^. In brief, emulsion samples were diluted with 2 wt.% Tween 20 to a range of 0.5–4 g oil/L. Next, the droplets were broken up in smaller droplets by sonication with the Branson Sonifier SFX550 (Brookfield, CT, USA), equipped with a 1/8 inch tapered microtip (Branson, Brookfield, CT, USA), at an amplitude of 35% for 15 s. This was done to increase the oil–water interface for effective hydrolysis of triglycerides and thereby to obtain accurate results. The droplet size distribution of the broken droplets was independent of the initial droplet size, as shown previously^36^. Next, about 20 µL of sample were weighed into a 2-mL microtube, and 1 mL of assay reagent was added. The samples were incubated in a temperature-controlled block, in the dark, and shaken at 850 rpm, at 20 °C, for 20 min. The absorbance was measured at a wavelength of 500 nm. The TAG content was determined using a calibration curve of a standard TAG dispersion (0.5–4 g oil/L).

#### Droplet size

A small volume (3 µL) of sample was taken from the emulsions after the emulsion was adjusted to an oil concentration of 10 wt.% (see section ‘Emulsion preparation by microfluidics’). This sample was observed using a Carl Zeiss Axioscope A1 optical microscope (Carl Zeiss, Breda, the Netherlands) equipped with a camera (AxioCam Mrc5). The droplet size of at least 190 droplets per independently prepared emulsion was measured using a custom-written script in Matlab R2019b (Mathworks, Natick, MA, USA), which is appropriate when droplets are very monodisperse^54^. The droplet size was expressed as the droplet diameter (D_1,0_) and was calculated from the droplet circumference. Since the droplet volume scales with the droplet diameter to the power of 3, a small systematic error leads to a much larger error in the productivity; therefore, as a check, the centre-to-centre distance of clustered droplets was determined, and with that a correction factor was calculated. This method was shown to be effective in previous work^36^.

#### Unadsorbed surfactant concentration

To ensure that the concentration of non-adsorbed Tween in the continuous phase was virtually independent of the oil droplet size, the amount of excess Tween 20 present in the continuous phase was estimated using a procedure that was based on previous work^4^: first, the number of droplets (*N*) with a certain droplet radius (*r*, m) was calculated per kg of oil:

7 $$N=\frac{1}{{\rho }_{{\text{oil}}}}\cdot \frac{1}{\frac{4}{3}\pi {r}^{3}}$$

where ${\rho }_{{\text{oil}}}$ is the density of the oil (920 kg/m^3^). The amount of adsorbed surfactant (${S}_{{\text{ad}}}$, in g per kg oil) was calculated using Eq. 8:

8 $${S}_{{\text{ad}}}=\frac{N 4\uppi {r}^{2} \Gamma }{1000}$$

where $\Gamma$ (mg/m^2^) is the specific adsorbed amount of surfactant at the oil–water interface. For Tween 20, different values for $\Gamma$ have been reported in literature, and we chose a relatively high value of 2.3 mg/m^2^ to estimate what the maximum amount of adsorbed surfactant could be^4,48^. Finally, the excess concentration of Tween 20 in the continuous phase (${C}_{{\text{excess}}}$, g/L) was calculated by:

9 $${C}_{{\text{excess}}}={C}_{{\text{added}}}-{S}_{{\text{ad}}} \cdot {\phi }_{{\text{oil}}} \cdot {\rho }_{{\text{cont}}}$$

where ${C}_{{\text{added}}}$ is the concentration of surfactant in the continuous phase (in g/L), ${\phi }_{{\text{oil}}}$ the mass ratio of oil to continuous phase (10/90 = 0.111), and ${\rho }_{{\text{cont}}}$ the continuous phase density, which was assumed to be 1000 kg/m^3^.

### Oxidation experiments

#### Oxygen measurement

The headspace oxygen content was monitored with a MOCON OpTech-O2 oxygen sensor (Ametek Mocon, Brooklyn Park, MN, USA) as previously described^39^. The initial amount of total available oxygen was calculated using a 1.35-mL headspace volume with 20.9% O_2_, 46.8 mg/kg oxygen concentration in the oil^55^, and 8.1 mg/kg oxygen concentration in the continuous phase^56^. The oxygen content was determined in four samples per independently prepared emulsion. Each sample was measured twice daily with three technical replicates, i.e., 12 measurements per datapoint. In addition, as preliminary results suggested that the tubes may have not been completely gas-tight, the oxygen ingress into the containers was determined. Hereto, a 1.5-mL tube was filled with 400 µL water, the headspace was flushed with N_2_, and subsequently the oxygen headspace concentration was measured over time.

#### Lipid extraction

Oil was extracted by adding 2.75 mL of 3:1 v/v hexane–isopropanol solvent mixture to 0.4 mL of emulsion^57^. The mixture was thoroughly vortexed and centrifuged (5000 × *g*, 20 °C, 20 min), and the upper layer, containing the hexane and extracted lipids, was carefully separated from the bottom layer. The hexane was evaporated under a stream of nitrogen at 25 °C until constant weight, and the remaining oil was stored at − 80 °C for 48 h to three weeks before further measurements were performed.

#### Lipid oxidation measurements by ^1^H NMR

Lipid hydroperoxides (primary oxidation products) and aldehydes (secondary oxidation products) were quantified simultaneously by ^1^H NMR spectroscopy, as previously described^39,58^. In brief, around 570 µL dry 5:1 CDCl_3_/DMSO‑*d*_6_ were added to a total of 30 µL extracted oil (section above) and transferred to 5-mm NMR tubes (Bruker, Billerica, MA, USA). Samples were measured on an Avance III 600 MHz spectrometer (Bruker BioSpin, Switzerland) equipped with a 5-mm cryo-probe at 295 K. Lipid hydroperoxides and aldehydes were quantified on a molecular substructure level as previously described using a single pulse and a band selective ^1^H NMR experiment^58^.

Epoxides (secondary oxidation products) were quantified using ^1^H-^13^C heteronuclear single quantum coherence (HSQC) NMR spectroscopy, which was based on previous work^39^. Here, the protocol was modified to improve the spectral resolution. 2D ^1^H-^13^C HSQC spectra with a band-selective ^13^C REBURB pulse were recorded using the standard “shsqcetgpsisp2.2” Bruker pulse sequence. In the ^13^C-dimension, a spectral width of 40 ppm with an offset of 58 ppm (*δ*_*C*_ 78–38 ppm) was used with 200 increments. In the ^1^H-dimension, the spectral width was 4 ppm with an off-set of *δ* 3 ppm (*δ*_*H*_ 5–1 ppm) with 1024 increments. Eight scans were collected by using a recycle delay of 0.5 s. For the ^13^C-dimension, zero-filling up to 1024 points was applied prior to Fourier transformation. The squared cosine (SSB 2) and Gaussian (LB − 0.2 Hz, GB 5 Hz) window functions were used for the ^13^C and ^1^H-dimensions, respectively. The same phasing was used for all samples. Baseline correction was performed automatically, and the upfield TG backbone peak was calibrated to *δ*_*H*_ 4.13 ppm and *δ*_*C*_ 61.9 ppm. The quantification of epoxides was based on automatic integration of predefined regions as previously reported^39^.

The spectral processing and integrations for the hydroperoxides, aldehydes, and epoxides was automatically done using MestReNova v14.1 (Mestrelab Research, S.L., Santiago de Compostela, Spain). The molecular substructures of the lipid hydroperoxides and epoxides were used to classify the fatty acid precursor of an oxidation product.

### Kinetic modelling

The data of lipid oxidation products formed and oxygen consumed in time were compared with a recently developed kinetic model^38^. This model was used previously to describe lipid oxidation for emulsions that were stabilised by different emulsifiers. For this research, the model was extended to incorporate the contribution of Tween 20 oxidation, and oxygen diffusion into the tubes. Additional information about how the model was used to describe experimental data is described in the Supporting Information (Supporting information section Introduction).

### Experimental design

Two emulsions were prepared independently for each droplet size (in total six emulsions). Per timepoint, two aliquots of emulsion were taken, lipids were extracted, and lipid oxidation products were quantified in each aliquot individually. The headspace oxygen concentration was measured as described earlier.

### Supplementary Information

Supplementary Information.

## Data Availability

The datasets generated during and/or analysed during the current study are available from the corresponding author on reasonable request.
