# Supplementary material for: Unravelling the effect of droplet size on lipid oxidation in O/W emulsions by using microfluidics
Source: Sci Rep. 2024 Apr 17;14:8895. doi: 10.1038/s41598-024-59170-9 (PMC11024159; doi:10.1038/s41598-024-59170-9)
Supplement: Supplementary file 1 — Supplementary Information. [file 41598_2024_59170_MOESM1_ESM.pdf]

Supplementary Information

Unravelling the effect of droplet size on lipid oxidation in O/W emulsions by using microfluidics

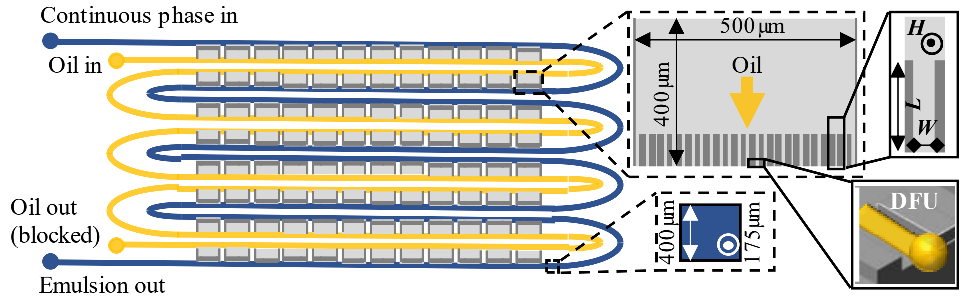

Figure S1. Top-view design of the Upscaled Partitioned EDGE chips used in this research. The blue 'twisted road' channel is the continuous phase channel, and the yellow 'twisted road' channel is the to-be-dispersed phase channel. The grey rectangular areas in between these channels are the main plateaus that contain the micro-plateaus with the droplet formation units (DFU). A 3D representation of a DFU is shown in the right lower corner, showing oil – in yellow –, being pushed out of the DFU and forming a droplet ready to detach. This illustration is not to scale, only 12 out of the 42 main plateaus are shown per row.

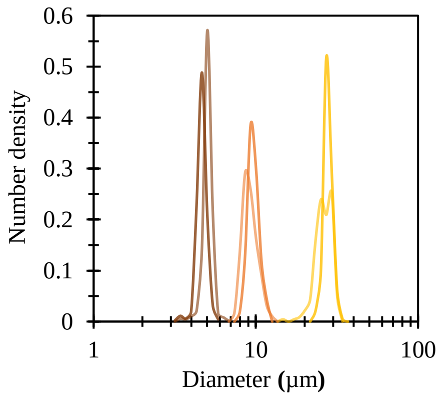

Figure S2. Droplet size distributions (number density as a function of droplet size) determined by using light microscopy of the six independently prepared emulsions. From left to right, the small (brown), intermediate (orange), and large (yellow) droplets.

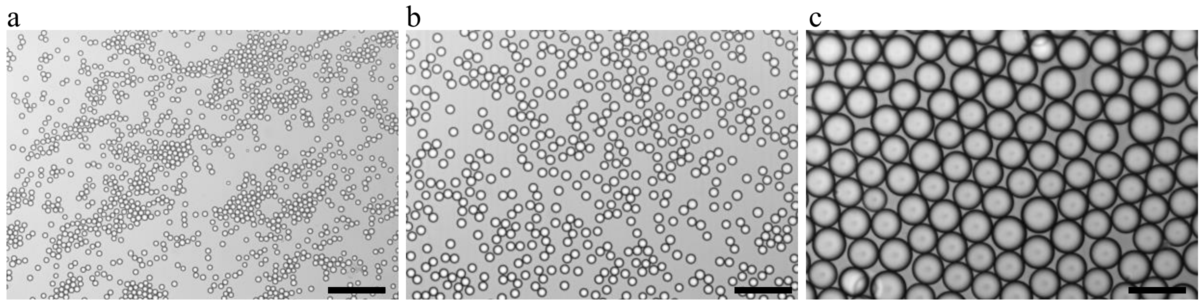

Figure S3. Light microscopy images of the rapeseed oil droplets in a continuous phase containing 2 wt.% Tween 20, which were prepared with microfluidics. Three different droplet sizes were prepared: small (a), intermediate (b), and large droplets (c). Droplet size distributions are shown in Figure S2. Light microscopy images of the other replicate emulsions are shown in Figure 1. Scale bar represents 50 µm.

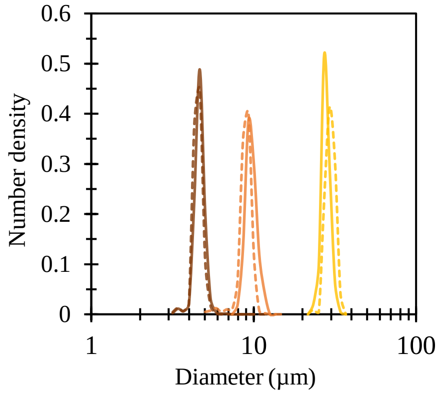

Figure S4. Droplet size distributions (number density as a function of droplet size) determined by using light microscopy directly after emulsification (solid lines) and after > 7 days of incubation (dashed lines). From left to right, the small (brown), intermediate (orange), and large (yellow) droplets.

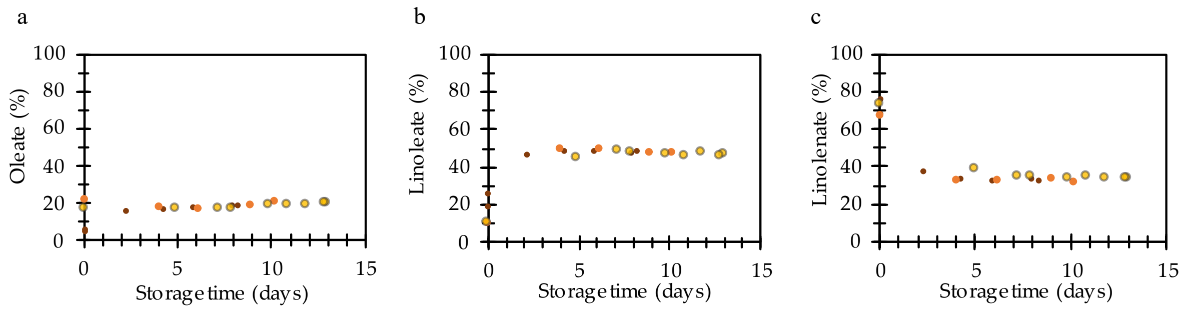

Figure S5. The contribution of different unsaturated fatty acid substrates to the total amount of oxidised products: oleate (a), linoleate (b), and linolenate (c). All oxidation products (lipid hydroperoxides, aldehydes, and epoxides) were summed for each fatty acid individually and divided by the total sum of oxidation products. Symbols correspond to emulsions with different droplet sizes: small droplets (D1,0 = 4.7 µm) (small, brown circles), intermediate droplets (D1,0 = 9.1 µm) (intermediate, orange, circles), and large droplets (D1,0 = 26.0 µm) (yellow, large circles).

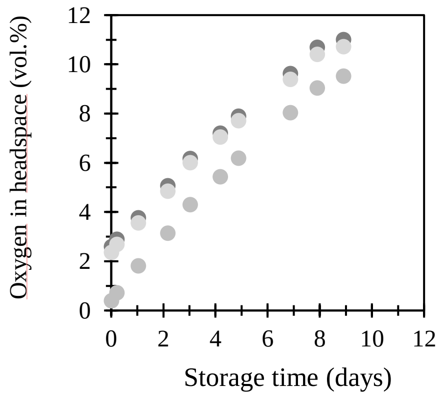

Figure S6. Oxygen (vol.%) in the headspace over incubation of 1.5-mL microcentrifuge tubes containing 400 µL of ultrapure water, which were flushed with nitrogen prior to incubation. The incubation was performed in the same way as for emulsion samples (section 2.2 'Incubation and sample taking'). Different coloured markers represent independently incubated tubes.

1. Reaction kinetic model

The lines in Figure 2 were related to the following lipid oxidation reactions, as proposed in:[46]

$$\mathrm{LH} \rightarrow \mathrm{L}^{\bullet} \tag{A I}$$

$$\mathrm{L}^{\bullet} + \mathrm{O}_2 \rightarrow \mathrm{LOO}^{\bullet} \tag{A II}$$

$$\mathrm{LOO}^{\bullet} + \mathrm{LH} \rightarrow \mathrm{LOOH} + \mathrm{L}^{\bullet} \tag{A III}$$

$$\mathrm{LOOH} \rightarrow \mathrm{LO}^{\bullet} \rightarrow \mathrm{LO} \tag{A IV}$$

$$\mathrm{LOOH} \rightarrow \mathrm{LOO}^{\bullet} \tag{A V}$$

Based on these reactions, first order reaction rate equations were derived as presented next, and shown in conjunction in Figure S7:

$$\frac{d[\mathrm{L}^{\bullet}]}{dt} = k_1 \cdot [\mathrm{LH}] - k_2 \cdot [\mathrm{L}^{\bullet}] \cdot [\mathrm{O}_2] + k_3 \cdot [\mathrm{LOO}^{\bullet}] \cdot [\mathrm{LH}'] \tag{A1}$$

$$\frac{d[\mathrm{LOO}^{\bullet}]}{dt} = k_2 \cdot [\mathrm{L}^{\bullet}] \cdot [\mathrm{O}_2] - k_3 \cdot [\mathrm{LOO}^{\bullet}] \cdot [\mathrm{LH}'] + k_5 \cdot [\mathrm{LOOH}] \tag{A2}$$

$$\frac{d[\mathrm{LOOH}]}{dt} = k_3 \cdot [\mathrm{LOO}^{\bullet}] \cdot [\mathrm{LH}'] - k_5 \cdot [\mathrm{LOOH}] \tag{A3}$$

$$\frac{d[\mathrm{LO}]}{dt} = k_4 \cdot [\mathrm{LOOH}] \tag{A4}$$

In line with the approach, a rate equation for the oxidation of Tween was added:

$$\frac{d[\mathrm{Tween}]}{dt} = k_{Tween} \cdot [\mathrm{Tween}] \cdot [\mathrm{O}_2] \tag{A5}$$

Finally, for oxygen diffusion, Fick's law was used. The measured diffusion coefficient was 2 mmol/h at maximum oxygen gradient (i.e., full headspace depletion inside the incubation container and 20.9 % outside). When adding this term, the rate equation for oxygen becomes:

$$\frac{d[\mathrm{O}_2]}{dt} = -k_{Tween} \cdot [\mathrm{Tween}] \cdot [\mathrm{O}_2] - k_2 \cdot [\mathrm{L}^{\bullet}] \cdot [\mathrm{O}_2] + ([\mathrm{O}_2]_i - [\mathrm{O}_2]_t) \cdot 0.002 \tag{A6}$$

where $[O_2]_i$ and $[O_2]_t$ are the initial molar concentration of oxygen (295 mmol/kg oil) and the molar concentration of oxygen at time point t, respectively.

The k-values used in the model to describe our data points were kept as close as possible to the ones reported previously because some of the applied conditions were similar: we used the same emulsifier (Tween 20), the same incubation temperature (25 °C), and the same concentration of iron-EDTA (oxidation initiator) in the continuous phase.[46,47] There were also experimental differences that are summarised in Table S2. Although it is not clear at the moment of writing what the underlying mechanisms are, we have tried to position the model lines as close as possible to the data points based on visual inspection, varying

initial radical concentration ($[L^•]_{t0}$), k_4 (secondary oxidation product formation), k_5 (radical formation by hydroperoxides), and introduce k_{Tween}. The values are summarised in Table S3.

(1) Starting with the largest droplets, we positioned the lines as close as possible to the data points.
   a. $[L^•]$ at t_0 was varied for two reasons: (i) the actual value cannot be measured, although the concentrations are expected to be low, and (ii), previously, it was found that within 1-25 μM the model described the data in similar fashion.[46]
   b. The k_4-value was adjusted because previously the hexanal and propanal concentrations were measured,[46] whereas here the total epoxide and aldehyde content was measured.
   c. The k_5-value was allowed to vary between emulsions. It is a reaction between iron and a lipid hydroperoxide, which is expected to increase with specific interfacial area (Table S2).[46]
   d. The Tween 20 oxidation reaction was included in our model because the weight-based ratio of Tween 20 to oil was ~ 15 times higher than in the experiments for which the original model was derived (Table S2).[46,47] Therefore, Tween 20 oxidation was probably negligible compared to lipid oxidation in the experiment for which the model was derived. That is not the case in our work, and since Tween 20 has been described to be sensitive to oxidation we included this reaction (Equation 11) and derived k_{Tween}-values.

(2) Taking the values obtained for the largest droplets as a starting point, the $[L^•]_{t0}$, k_5-value, and k_{Tween}-value were changed to position the model lines as close as possible to the data for both the small- and intermediate droplets (Figure 2, Table S3). There seems to be a connection between the variation of the parameters and the droplet size, which indicates that the effect of droplet size on lipid oxidation is systematic, which is part of follow up research.

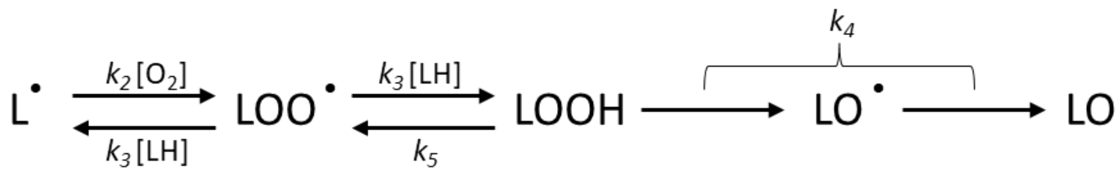

Figure S7. Overview of reactions incorporated in the model (Schroën et al., 2022a): $L^•$ is a carbon radical, $LOO^•$ peroxyl radical, LOOH hydroperoxide (primary oxidation product), $LO^•$ alkoxy radical, LO secondary oxidation products, and O_2 oxygen, which was also measured.

Table S1. Calculation of the ratio of interfacial Tween 20 to total Tween 20 for the small droplets ($D_{1,0}$ = 4.7 µm).

Diameter smallest droplets (m)	$4.5 \cdot 10^{-6}$
Volume of oil per L emulsion (L)	0.1
Volume of one droplet (m^3)	$4.8 \cdot 10^{-17}$
Number of droplets	$2.1 \cdot 10^{12}$
Interface one droplet (m^2)	$6.4 \cdot 10^{-11}$
Total interface per L emulsion (m^2)	133
Tween 20 interfacial load (mg/m^2)	2.3
Tween 20 at oil droplets interface per L emulsion (g)	0.2
Tween 20 in emulsion (g/L)	18
Fraction of interfacial Tween 20 (compared to total amount)	1.9%

Table S2. Differences in kinetic model parameters between our study and the modelling study [46] (in which the data from [47] were used).

	**Tween 20 emulsion in (Schroën et al., 2022)**	**This research**
**Droplet size (μm)**	1.4	4.7, 9.1, 26
**Tween 20 in continuous phase (g/L)**	~ 0.5	~ 20
**Tween 20 in emulsion (wt.%)**	0.35	1.8
**Oil concentration (wt.%)**	30	10
**Hydroperoxide content t_0 (mmol/kg oil)**	~ 10	1
**O_2 content initially available (mmol/kg oil)**	158	295
**LH' content (mmol/kg oil)**	4510	3990
**Hydroperoxides measured**	Conjugated dienes	Total hydroperoxides
**Secondary oxidation products measured**	Propanal, hexanal	Total aldehydes and epoxides

Table S3. Differences in parameters for the kinetic model between our study and the modelling study [46] (in which the data from [47] was used). '-' indicates that this parameter was not used.

**Parameter**	**(Schroën et al., 2022)**	**Small droplets**	**Intermediate droplets**	**Large droplets**
k_1	-	-	-	-
k_2	19.5	19.5	19.5	19.5
k_3	1.95	1.95	1.95	1.95
k_4	$6.0 \cdot 10^{-3}$	$2.0 \cdot 10^{-3}$	$2.0 \cdot 10^{-3}$	$2.0 \cdot 10^{-3}$
k_5	$3.6 \cdot 10^{-3}$	$3.8 \cdot 10^{-4}$	$3.4 \cdot 10^{-4}$	$2.4 \cdot 10^{-4}$
k_{Tween}	-	$4.0 \cdot 10^{-3}$	$2.8 \cdot 10^{-3}$	$1.5 \cdot 10^{-3}$
$[L\cdot]_{t0}$	$1.0 \cdot 10^{-6}$	$3.5 \cdot 10^{-5}$	$2.0 \cdot 10^{-5}$	$1.0 \cdot 10^{-5}$
